# A broad-range survey of ticks from livestock in Northern Xinjiang: changes in tick distribution and the isolation of *Borrelia burgdorferi sensu stricto*

**DOI:** 10.1186/s13071-015-1021-0

**Published:** 2015-09-04

**Authors:** Yuan-Zhi Wang, Lu-Meng Mu, Ke Zhang, Mei-Hua Yang, Lin Zhang, Jing-Yun Du, Zhi-Qiang Liu, Yong-Xiang Li, Wei-Hua Lu, Chuang-Fu Chen, Yan Wang, Rong-Gui Chen, Jun Xu, Li Yuan, Wan-Jiang Zhang, Wei-Ze Zuo, Ren-Fu Shao

**Affiliations:** School of Medicine, Shihezi University, 832003 Shihezi, Xinjiang Peoples Republic of China; Institute of Agricultural and Rural Development, Pingdingshan University, 467000 Pingdingshan, Henan Peoples Republic of China; School of Agriculture, Shihezi University, 832003 Shihezi, Xinjiang Peoples Republic of China; Jinan Center for Disease Control and Prevention, 250021 Jinan, Shandong Peoples Republic of China; School of Animal & Science, Shihezi University, 832003 Shihezi, Xinjiang Peoples Republic of China; Veterinary Research Institute, Xinjiang Academy of Animal Sciences, 830000 Urumqi, Xinjiang Peoples Republic of China; Ili Center of Animal Disease Control and Diagnosis, 835000 Ili, Xinjiang Peoples Republic of China; Xijiang Entry - Exit Inspection and Quarantine Authority of the PRC, 830063 Urumqi, Xinjiang Peoples Republic of China; First Affiliated Hospital of School of Medicine, Shihezi University, 832008 Shihezi, Xinjiang Peoples Republic of China; Genecology Research Center, University of the Sunshine Coast, Queensland, 4558 Australia

**Keywords:** Tick species, *Borrelia burgdorferi*, Northern Xinjiang

## Abstract

**Background:**

Borreliosis is highly prevalent in Xinjiang Uygur Autonomous Region, China. However, little is known about the presence of *Borrelia* pathogens in tick species in this region, in addition *Borrelia* pathogens have not been isolated from domestic animals.

**Methods:**

We collected adult ticks from domestic animals at 19 sampling sites in 14 counties in northern Xinjiang from 2012 to 2014. Ticks were identified to species by morphology and were molecularly analysed by sequences of mitochondrial *16S rDNA* gene; 4–8 ticks of each species at every sampling site were sequenced. 112 live adult ticks were selected for each species in every county, and were used to culture *Borrelia* pathogens; the genotypes were then determined by sequences of the *5S-23S rRNA* intergenic spacer and the outer surface protein A (*ospA*) gene.

**Results:**

A total of 5257 adult ticks, belonging to four genera and seven species, were collected. Compared with three decades ago, the abundance of the five common tick species during the peak ixodid tick season has changed. Certain tick species, such as *Rhipicephalus turanicus* (*Rh. turanicus*), was found at Jimusaer, Yining, Fukang, and Chabuchaer Counties for the first time. Additionally, the sequence analyses showed that the *Hyalomma asiaticum* (*Hy. asiaticum*), *Haemaphysalis punctata* (*Ha. punctata*), and *Dermacentor marginatus* (*D. marginatus*) that were collected from different sampling sites (≥3 sites) shared identical *16S rDNA* sequences respectively. For the tick species that were collected from the same county, such as *Hy. asiaticum* from Shihezi County and *Rh. turanicus* from Yining County, their 16S *rDNA* sequences showed genetic diversity. In addition, sixteen *Borrelia* isolates were found in *Hy. asiaticum*, *Ha. punctata*, *D. marginatus* and *Rh. turanicus*, which infested cattle, sheep, horse and camel in Yining, Chabuchaer, Shihezi and Shawan Counties. All of the isolates were genetically identified as *B. Burgdorferi sensu stricto*.

**Conclusions:**

Warmer and wetter climate may have contributed to the altered distribution and abundance of the five most common ticks in northern Xinjiang. The genetic analyses showed that certain tick species, such as *Hy. asiaticum* or *Rh. turanicus*, exhibit genetic commonness or diversity. Additionally, this study is the first to isolate *B. burgdorferi sensu stricto* in *Hy. asiaticum asiaticum*, *H. punctata*, *D. nuttalli* and *D. marginatus* ticks from domestic animals. These ticks may transmit borreliosis among livestock.

**Electronic supplementary material:**

The online version of this article (doi:10.1186/s13071-015-1021-0) contains supplementary material, which is available to authorized users.

## Background

Lyme disease (borreliosis) is one of the most prevalent tick-borne zoonoses in eastern Asia [1]. One of its aetiologic agents, *Borrelia burgdorferi sensu lato*, is primarily distributed in northern China, whereas *B. garinii* and *B. afzelii* are distributed in northeastern and northwestern China [2]. In 2001, *B. garinii* and *B. afzelii* were firstly isolated from free-living *Ixodes persulcatus* in Xinjiang, China (XARC) [3]. Liu et al. reported that the blood samples of domestic animals in Xinjiang were highly positive (14 %) to *Borrelia burgdorferi*-like bacteria, as determined by polymerase chain reaction (PCR) [4], but the pathogens have not been isolated from ticks infecting livestock.

Xinjiang occupies one-sixth of China and borders eight countries with a 5,600-km long borderline. There are three major mountains in Xinjiang: Altay Mountain in the north, Tianshan Mountain in the middle, and Kunlun Mountain in the south. Between the mountains are two large basins, Junggar Basin in the north and Tarim Basin in the south [5]. High mountains, valleys, plains, Gobi desert, and other additional characteristics constitute the various landscapes of northern Xinjiang [6]. Additionally, this region is halfway along the old Silk Road between eastern Asia and Europe, and international livestock trade is frequent [3].

Forty-two species of ticks in nine genera have been identified in Xinjiang, which represent more than 1/3 of the total tick species found in China [7]. According to Kong *et al.*, *Ixodes persulcatus* (*I. persulcatus*), *Dermacentor nuttalli* (*D. nuttalli*), *Hy. asiaticum asiaticum*, *D. marginatus*, and *Dermacentor niveus* (*D. niveus)* were the five dominant tick species during the peak ixodid tick season 30 years ago [8]. Since then, there have been few comprehensive reports regarding the distribution and abundance of ticks in northern Xinjiang.

In the present study, we investigated the distribution and abundance of ticks from livestock in northern Xinjiang. We also isolated *Borrelia* pathogens from ticks for the first time in this region.

## Methods

### Sampling area

**Fig. 1:**
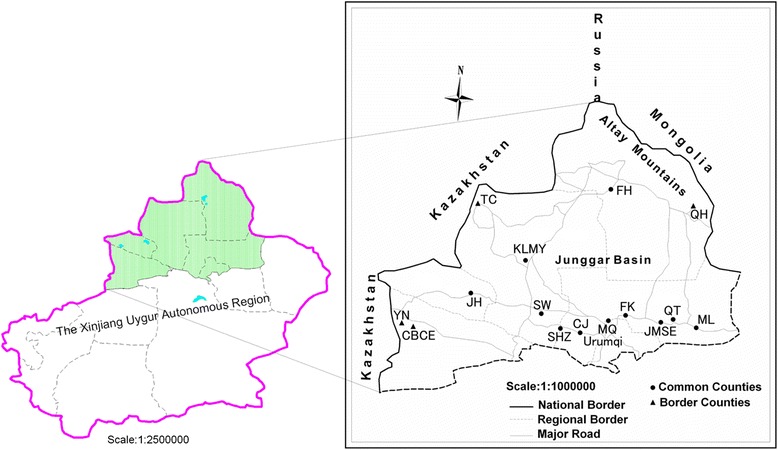
A map of the study area. Left: the Xinjiang Uygur Autonomous Region. Right: the 14 surveyed counties in northern Xinjiang

**Fig. 2:**
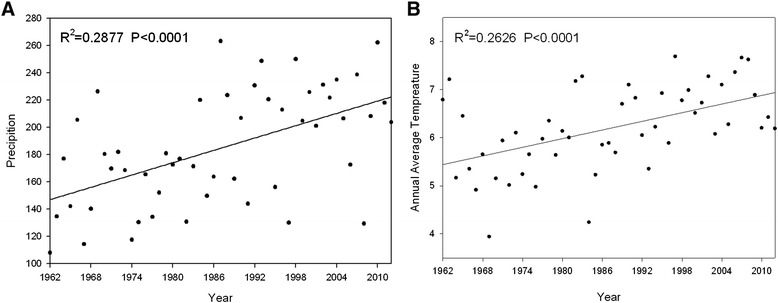
The annual precipitation and average annual temperatures in northern Xinjiang from 1962–2012. The annual precipitation and average annual temperature are shown in (**a**) and (**b)**, respectively. All the data came from eight international surveillance sites (Jinghe, Qitai, Altay, Fuyun, Hefeng, Karamay, Yining, and Urumqi) in northern Xinjiang and were collected from 1962–2012. The raw data are from the China Meteorological Data Sharing Service System (http://cdc.cma.gov.cn/) and were analyzed using Sigma Plot software

We established 19 sampling sites in 14 counties in northern Xinjiang–Jimusaer, Qitai, Mulei, Fukang, Miquan, Chanagji, Shihezi, Shawan, Karamay, Fuhai, Qinghe, Tacheng, Yining and Chabuchaer, from 2012 to 2014, in May when the peak activities of adult ticks occur in northern Xinjiang. The first 10 counties are primarily livestock husbandry regions, and the 4 remaining counties include 4 trade ports (Takesh Ken, Baketu, Yining, and Dulata) that are adjacent to Mongolia and Kazakhstan and are important trade zones for livestock and livestock products (Fig. 1). In accordance with the different types of landscapes, 1–4 sampling sites were selected in each county. Geographic information regarding the tick habitats and the geographic coordinates, altitude, and plant distributions of all 33 sampling sites were shown in Additional file 1: Table S1. The annual precipitation and average annual temperatures from 1962 to 2012 were shown in Fig. 2.

The livestock reared in these regions are mainly autochthonous. The vast majorities (approximately 95 %) of the sheep are Kazakh, Chinese Merino, Hu, and Altay breeds; the majority of the cattle are Xinjiang Brown, Holstein and Kazakh breeds. The horses are the Kazakh breed and the camels are the Bactrian breed. All of the livestock we checked for ticks were grazed in the traditional manner; no chemical acaricides were used prior to the tick sampling.

### Tick sampling and identification

Adult ticks were collected over three consecutive years. In 2012, the ticks were collected from 22 livestock flocks at 12 sampling sites in 10 counties. In 2013, seven livestock flocks at seven sampling sites in 5 counties were surveyed; these sampling sites are in various landscapes. In 2014, four livestock flocks in four counties were surveyed. The information regarding all of the collected specimens, including their location, host, number of ticks collected from the body of each animal and the date of collection, were recorded. The tick sampling was performed over the entire body of each animal, including sheep, cattle, horses, and camels, at different intervals, and the collected ticks were kept alive until they were transferred to the laboratory. The ticks collected were counted, and identified to species by morphology according to Walker et al. [9] and Estrada-Peňa et al. [10].

### Genetic analysis

After detailed morphological investigation, 110 representative tick specimens, with 1–4 ticks for each tick species at every sampling site, were used to analyse the genetic diversity. The genomic DNA extraction was performed using a commercial kit (DNeasy Blood & Tissue Kit, Qiagen GmbH, Hilden, Germany) according to the manufacturer’s instructions. The *16S rDNA* mitochondrial gene sequences were then amplified according to the protocol of Black et al. [11]. The PCR products were purified using the TIANgel Midi Purification Kit (TIANGEN, Beijing, China) and then sequenced.

### Isolation and identification of *Borrelia* pathogens

A total of 112 live adult ticks, including 34 *Hy. Asiaticum asiaticum*, 39 *H. punctata*, 10 *D. nuttalli*, 8 *D. marginatus*, and 21 *Rh. turanicus* specimens, were collected from each county and were selected for the isolation and identification of *Borrelia*. The ticks were first dipped in 70 % alcohol for approximately 1 h and dissected on sterile plates according to a method described by Burgdorfer et al. [12]. The midguts of each tick species from a county were then transferred to a tube containing 6 mL of BSK-H medium (Nunc, Roskilde, Denmark). All of the cultures were then incubated at 33 °C for up to 6 weeks and examined periodically using silver staining. Twenty-four samples were also photographed using a microscope (Model: BX60, OLYMPUS, Japan) equipped with a digital camera (Model: DP70, OLYMPUS, Japan) linked to a computer. Additionally, to determine the *Borrelia* genotype, the sequences of *5S-23S rRNA* intergenic spacers and the outer surface protein A (OspA) gene were amplified and sequenced from positive cultures.

### Cycling conditions for the genetic analyses of the 110 representative tick specimens and the *Borrelia* isolates

The detailed cycling conditions for 16S *rDNA*, the *5S-23S rRNA* intergenic spacer, and *OspA* are described in the Additional file 2.

### Phylogenic analysis

Data comparisons were performed using those tick species that had more than 10 available specimens. Sequence alignments to determine the nucleotide percentage from each species were performed using Clustal X 2.0 [13], and the phylogenic relationships among the representative tick specimens were inferred using MEGA5 [14]. A phylogenic tree was then constructed using the neighbor-joining method [15].

A total of 67 reference sequences, including sequences from the *Dermanyssus gallinae* (L34326.1) and *Spinturnix myoti* (FJ225960) outgroups, were used for the phylogenetic analysis, and 23 nucleotide sequences from our study have been deposited in the GenBank database (16S *rDNA*: KF547980, KF547981, KF547982, KF547983, KF547984, KF547985, KF547986, KF547987, KF547988, KF547989, KF547990, KF547991, KF547992, KF547993, KF547994, and KF547995; *5S-23S rDNA* intergenic spacer of *Borrelia*: KF547996, KF547997, KF547998, KJ459337, KJ459338, KJ459339 and KJ459340).

## Results

### Tick collection and morphological identification

A total of 5257 adult ticks, from seven species and four genera, were collected from 19 sampling sites in 14 counties in northern Xinjiang (Table 1). *Hy. asiaticum asiaticum* (32.70 %) was the most frequently collected species, followed by *Ha. punctata* (31.17 %), *D. nuttalli* (13.47 %), *D. marginatus* (12.10 %), *Rh. turanicus* (9.53 %), *Hyalomma detritum* (0.02 %), and *Haemaphysalis concinna* (0.02 %) (Table 1).

**Table 1:** The counts of each tick species from the 19 sampling sites between 2012 and 2014

Counties	Flock NO. (year)	Hosts	*Haemaphysalis*		*Dermacentor*		*Hyalomma*		*Rhipicephalus*
			*H. punctata* (♂/♀)	*H. conicinna* (♂/♀)	*D. marginatus* (♂/♀)	*D. nuttalli* (♂/♀)	*H. asiaticum* (♂/♀)	*H. detritum* (♂/♀)	*R. turanicus* (♂/♀)
Chabuchaer (CBCE)	1#(2013)	Sheep							151 (77/74)
	2#(2013)	Sheep							191 (97/94)
	3#(2014)	Sheep							69 (32/37)
Changji (CJ)	1#(2012)	Cattle					180 (76/104)		
Fuhai (FH)	1#(2012)	Sheep	11 (5/6)			15 (6/9)	148 (95/53)		
	2#(2012)	Sheep	9 (4/5)			8 (3/5)	150 (90/60)		
Fukang (FK)	1#(2012)	Cattle, sheep			9 (6/3)		60 (43/27)		16 (12/4)
	2#(2012)	Cattle, sheep	152 (87/65)		161 (80/81)				
	3#(2012)	Cattle, sheep					139 (86/53)		
Jimusaer (JMSE)	1#(2012)	Sheep	76 (54/22)						
	2#(2012)	Cattle, sheep				195 (104/91)	22 (8/14)		
	3#(2012)	Camel							74 (11/63)
Karamay (KLMY)	1#(2012)	Cattle					80 (40/40)		
	2#(2012)	Cattle	89 (22/67)						
Miquan (MQ)	1#(2012)	Cattle, sheep					93 (36/57)		
	2#(2012)	Cattle, sheep			90(70/20)		112 (48/64)		
Mulei (ML)	1#(2012)	Cattle, sheep	38 (32/6)			47 (44/3)		1 (0/1)	
	2#(2012)	Cattle, sheep				97 (65/32)			
	3#(2012)	Cattle, sheep				196 (112/84)			
Qinghe (QH)	1#(2012)	Sheep	62 (41/21)						
	2#(2014)	Sheep	41 (18/23)				136 (66/70)		
Qitai (QT)	1#(2012)	Cattle, sheep	187 (121/66)				219 (160/59)		
	2#(2012)	Sheep				22 (5/17)			
	3#(2012)	Cattle, sheep					48 (12/36)		
	4#(2012)	Sheep				65 (41/24)	29 (23/6)		
Shawan (SW)	1#(2012)	Sheep, horse	100/76		30/19		47/29		
	2#(2013)	Sheep	112 (63/49)		87 (57/30)		44 (25/19)		
	3#(2013)	Sheep	101 (62/39)		83 (55/28)		42 (25/17)		
Shihezi (SHZ)	1#(2013)	Cattle, sheep	187/150		40/27	21/14	57/35		
	2#(2014)	Camel, sheep	121 (72/49)		54 (31/23)		83 (49/34)		
Tacheng (TC)	1#(2013)	Sheep		0/1	23/13				
Yining (YN)	1#(2013)	Cattle, Sheep	48/58						
	2#(2014)	Cattle, Sheep	73 (32/41)						
Total number		5257	1691 (948/743)	1 (0/1)	636 (392/244)	708 (418/244)	1719 (961/758)	1 (0/1)	501 (229/272)
Percentage (%)			32.17 %	0.02 %	12.10 %	13.47 %	32.70 %	0.02 %	9.53 %

### Molecular identification and analysis

**Fig. 3:**
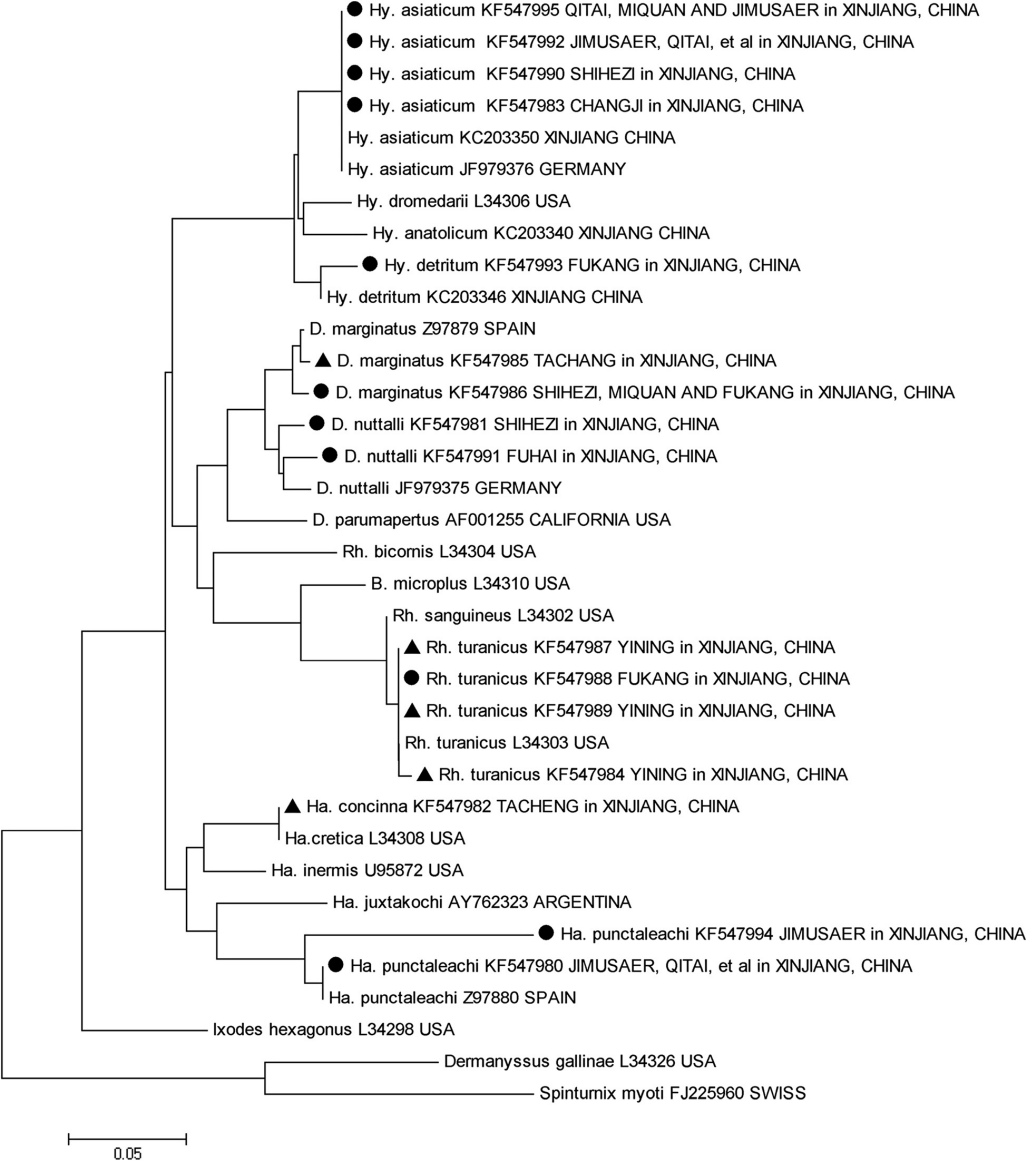
The phylogenic tree inferred from the *16S rDNA* sequences of the representative tick specimens. The evolutionary history was inferred using the neighbor-joining method. The new sequences provided by the present study are indicated by a black dot (ticks collected in internal counties) or a black triangle (ticks collected from border counties) in front of the sequence name (containing the accession number). The phylogenic analyses were conducted using MEGA5

A phylogenetic tree based on 16S *rDNA* sequences of the representative tick specimens is shown in Fig. 3. The results showed that *Hy. asiaticum*, *Ha. punctata*, and *D. marginatus* samples that were obtained from different sampling sites (≥3 sites) shared common 16S *rDNA* sequences. Their accession numbers are KF547992, KF547980, and KF547986, respectively. Additionally, the 16S *rDNA* sequences of certain tick species from the same sampling site showed diversity. For example, 3 different sequences were obtained from *Rh. turanicus* from Yining County, with the accession numbers KF547984, KF547987, and KF547989.

### Isolation and identification of *Borrelia* from ticks

**Fig. 4:**
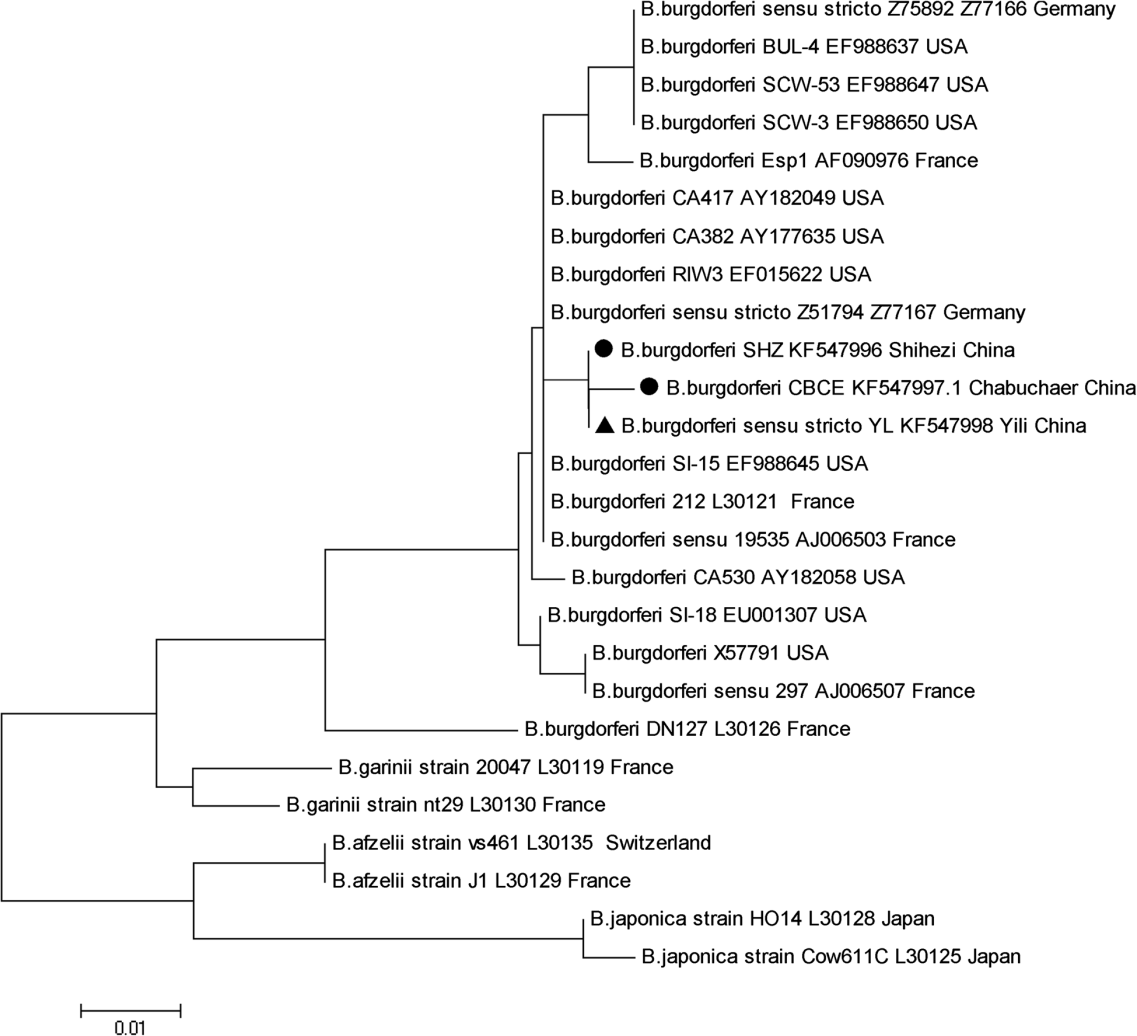
The phylogenic tree inferred from the *5S-23S rRNA* intergenic spacer sequences of the 16 *Borrelia burgdorferi* isolates. The evolutionary history was inferred using the neighbor-joining method. The phylogenic analyses were conducted using MEGA5

Sixteen positive cultures of *Borrelia* from *Hy. Asiaticum asiaticum*, *Ha. punctata*, *D. marginatus*, and *Rh. turanicus* collected in Yining, Chabuchaer, Shihezi and Shawan Counties were observed. Their *OspA* sequences showed 100 % identity to the *B. burgdorferi* reference B31 strain (Accession NO: X63412). Their *5S-23S rRNA* sequences showed that all of the positive culture extracts are *B. Burgdorferi sensu stricto* (Fig. 4).

## Discussion

The distribution of ticks within a specific habitat depends on several environmental and climatic factors, such as annual rainfall, atmospheric temperature and relative humidity (RH), vegetation cover, altitude and host availability. This study was conducted in May, from 2012 to 2014, when the RH and the atmospheric temperature provide an ideal environment for most tick species. Therefore, the abundance of adult ticks on livestock is expected to be at its peak. In the present study, the spatial distribution and abundance of ticks infesting livestock was assessed. The following phenomena were observed and compared with previous findings, especially with those from the investigation conducted by Kong et al. [8].

First, the abundance of *I. persulcatus* was reduced. In previous studies, *I. persulcatus* was found to be the dominant tick species in northern China, including Xinjiang, and the species was found distributed at altitudes of 1300–2120 m above sea level (*m.a.s.l*). In this study, *I. persulcatus* was not identified in the various analysed landscapes over a wide range of altitudes. When comparing the average monthly precipitation and temperatures from 1973 to 2012, we found that lower summer precipitation levels coinciding with raised summer temperatures may inhibit the survival and development of *I. persulcatus*.

Second, *Rh. turanicus* has a tendency to shift northwards. It is primarily distributed in the plains and rural areas of the Shanxi, Guangxi, Hainan and Xinjiang Provinces in China and is the second most abundant tick species in the southern region of Xinjiang [16]. However, *Rh. turanicus* was first collected in the plains of Jimusaer, Yining, Fukang, and Chabuchaer Counties, in northern Xinjiang. By tracing the movement routes of *Rh. turanicus*, we found that from 1973 to1985, the species was distributed below 25° N, which then shifted northwards to the hilly terrain of Huocheng County (44° N) in 1993 [17], and it is currently found in the semi-desert grasslands of Fukang County (48° N). This shift in the latitudinal distribution shows that the high temperatures in winter and the increased precipitation in spring and autumn may favour the survival, activity and development of *Rh. turanicus*. This phenomenon appears to be coincident with the spatial changes observed in *I. ricinus* in Europe due to the effects of climate change [18, 19].

Third, the geographical distribution of *H. punctata* and *D. marginatus* has expanded. In China, *H. punctata* is only distributed in Xinjiang and Gansu Province and ranges from 580 to 1700 *m.a.s.l*. Its habitat varies, including meadows, conifer-broadleaf forests, thickets, and semi-desert. In this study, *H. punctata* was found to be the second most abundant and the most widespread tick species compared with a report for the period from 1973 to 1985. We collected *H. punctata* from Jimusaer, Qitai, Mulei, Fukang, Karamay, Shihezi, Shawan, Fuhai, Qinghe, and Tacheng Counties. Additionally, in China, *D. marginatus* is distributed throughout Xinjiang and ranges from 300 to 1600 *m.a.s.l*. Its habitats include semi-desert, saline-alkali soil, grasslands and thickets. *D. marginatus* used to be scattered throughout Yining, Tacheng, Karamay and Aertai Countries; however, in our study, we collected the species in Fukang, Miquan, Shihezi and Shawan Counties. These results strongly suggest that *H. punctata* and *D. marginatus* have expanded their range in the last three decades, particularly into the northern parts of Xinjiang. More studies are needed to further understand the phenomena above. For example, the survival rates of the eggs, larvae, nymphs and adult ticks of *I. persulcatus*, *H. punctata* and *D. marginatus* should be assessed under different RHs and environmental temperatures.

The analyses of the mitochondrial *16S rDNA* sequences were not conclusive but did provide support regarding the genetic similarities or diversity of several of the special tick species in some of the regions, which may have resulted from geographic separation and historical international livestock trade. *Hy. asiaticum* and *Ha. punctata* from Jimasa, Qitai, Mulei, Fuhai, Shihezi, Shawan, Karamay, Qinghe, Tacheng and Chabuchaer were found to share common *16S rDNA* sequences (KF547992 and KF547980). These results show that these tick species are indigenous and evolved from the same lineage. In contrast, with the long-distance movement of livestock during periods of historical commercial trade, “foreign” ticks (tick species with different lineages) were introduced into some of the regions. Therefore, identical tick species from the same region showed genetic diversity, such as *Hy. asiaticum* from Shihezi County (KF547992 and KF547990) and *Rh. turanicus* from Yining County (KF547984, KF547987 and KF547989).

Additionally, a high prevalence of tick-borne diseases (TBDs), such as Crimean-Congo hemorrhagic fever, borreliosis, and tick-borne encephalitis, exists in Xinjiang, and some tick-borne pathogens have appeared in the adjacent countries [20–23].

Borreliosis, which is caused by *Borrelia burgdorferi sensu lato*, is an important endemic zoonosis with a distribution closely related to the major ixodid tick vectors [24, 25]. Of the 109 species of ticks identified in China, *B. burgdorferi* has been isolated from nine ixodid ticks, including *I. persulcatus*, *I. granulatus*, *I. acutitarsus*, *H. longicornis*, *H. bispinosa*, *H. concinna*, *H. formosensis*, *Boophilus microplus* and *D. silvarum* [26, 27]. We attempted to isolate *Borrelia* pathogens from the predominant tick species for the following reasons: i) borreliosisis prevalent in domestic animals in Xinjiang [4, 28]; ii) Liu et al. first detected 5 s-23 s rRNA intergenic spacer and flagllin gene of *B. burgdorferi sensu stricto* from livestock blood samples in the Ili and Altay Districts in northern Xinjiang in 2013; and iii) *I. persulcatus*, which is the vector for *B. burgdorferi*, has not been identified from the domestic animal species we sampled. Ultimately, we isolated *B. burgdorferi sensu stricto* from *Hy. Asiaticum asiaticum*, *H. punctata*, *D. marginatus* and *Rh. Turanicus* we collected from domestic animals. This result shows that *Hy. asiaticum*, *H. punctata*, *D. marginatus* and *Rh. turanicus* are carriers of *B. burgdorferi sensu stricto* and act as potential vectors for livestock borreliosis. Although our present research showed that *5S-23S rRNA* intergenic spacer sequence of *B. burgdorferi* was positively detected from *Hy. Asiaticum*, *H. punctata*, *D. marginatus* and *Rh. turanicus* that were infesting the domestic animals we sampled, future work, such as virulence tests of tick bites in animals and transmission experiments, should be performed to further confirm whether these tick species are vectors for *B. burgdorferi sensu stricto*. Moreover, the seroprevalence and level of bacterial isolation from domestic animals should be continuously monitored.

## Conclusions

In this study, we collected 5257 adult ticks from the sheep, cattle, horses and camels from 19 sampling sites in 14 counties in Xinjiang along the New Eurasian Continental Bridge, which is the area adjacent to Mongolia and Kazakhstan. The adult ticks belonged to four genera and seven species. We found that *Hy. Asiaticum asiaticum*, *H. punctata*, *D. nuttalli*, *D. marginatus*, and *R. turanicus* are the dominant tick species during the peak activity season of adult ticks in northern Xinjiang; this differs from the dominant tick species reported three decades ago. This change is likely due to the local climate change, which has become warmer and wetter. The phylogenetic tree indicates that certain tick species exhibit genetic similarities or diversity, which may be related to geographic separation and historical international livestock trade. Additionally, *B. burgdorferi sensu stricto* was isolated from *Hy. Asiaticum asiaticum, H. punctata, D. marginatus* and *Rh. turanicus* from domestic animals for the first time in northern Xinjiang*.* These four tick species may act as potential vectors for livestock borrelia infection in Xinjiang.

## Additional files

Additional file 1: Table S1. Geographic information for the 19 sampling sites at 14 surveyed counties. (DOC 55 kb) Additional file 2:
**PCR protocol for the detection of 110 representative tick specimens and Borrelia isolates, northern Xinjiang, China.** (DOC 50 kb)
