# Supplementary material for: A broad-range survey of ticks from livestock in Northern Xinjiang: changes in tick distribution and the isolation of Borrelia burgdorferi sensu stricto
Source: Parasit Vectors. 2015 Sep 4;8:449. doi: 10.1186/s13071-015-1021-0 (PMC4560164; doi:10.1186/s13071-015-1021-0)
Supplement: Additional file 1: Table S1. — Geographic information for the 19 sampling sites at 14 surveyed counties. (DOC 55 kb) [file 13071_2015_1021_MOESM1_ESM.doc]

| **County** | **Sampling sites** | **Tick habitats** | **Latitude and longitude** | **Altitude and plant distribution** |
| --- | --- | --- | --- | --- |
| Chabuchaer | Sunzaqi Township | Grasslands, wetlands and valley | 43°896859′N and 81°062009′E | 575 m.a.s.l. Diversiform-leaved Poplar, willow and rich vegetation. |
|  | Zhuohuoer Township | Grasslands and wetlands | 43°875802′N and 81°153338′E | 586 m.a.s.l. Diversiform-leaved poplar, willow and rich vegetation |
| Changji | Daxiqu Township | Mountains, plains and semi-desert | 43°107398′N and 87°256714′E | 529 m.a.s.l. *Haloxylonammodendron*, Tamarix, *Achnatherumsplendens*. |
| Fuhai | Fuhai Township | Semi-desert and Gobi | 47°112653′N and 87°459518′E | 496 m.a.s.l. Bush and narrow-leaved oleaster. |
|  | Jiereatele Township | Mountain meadow and grasslands | 47°103489′N and 87°448988′E | 495m.a.s.l. Diversiform-leaved poplar and narrow-leaved oleaster. |
| Fukang | Sangonghe Kazak Township | Forest, plains and lakes | 44°018860′N and 88°084473′E | 1079 m.a.s.l. Spruce, shrubs and rich vegetation. |
| Jimusaer | Qingyanghu Township | Hills and Gobi | 44°009547′N and 88°988642′E | 794 m.a.s.l. Shuttle, *Achnatherumsplendens*, wormwood |
| Karamay | Wuerhe Township | Semi-desert and the Yardang landform | 45°982431′N and 85°832434′E | 275 m.a.s.l. Reeds, Achnatherumsplendens, Populuseuphratica |
|  | Xiaoguai Township | Semi-desert and Gobi | 45°130874′N and 85°051303′E | 292 m.a.s.l. Haloxylonammodendron, Tamarix, reed, Achnatherumsplendens, Setariaviridis |
| Miquan | Boyanghe Kazak Township | Hills and plains | 43°955114′N and 87°899498′E | 956 m.a.s.l. Shuttle, *Achnatherumsplendens*, and sparse bush and short grass. |
| Mulei | Uzbek Township | High mountains and forest | 43°937856′N and 90°351224′E | 1071 m.a.s.l. Spruce, shrubs and rich vegetation. |
| Qinghe | Chaganguole Township | High mountain and grasslands | 46°457804′N and 90°776743′E | 1289 m.a.s.l. Herb plants, shrubs and subalpine meadow. |
| Qitai | Xidi Township | Gobi and oasis | 44°024203′N and 89°746333′E | 862 m.a.s.l. Shuttle, *Achnatherumsplendens*, wormwood |
| Shawan | Boertongute Pasture | Mountains, semi-desert and dunes | 43°987447′N and 85°361939′E | 1361 m.a.s.l. Conifer-broadleaf forest,riverside thickets and *Achnatherumsplendens*. |
|  | Niujuanzi Pasture | Mountain meadow, valley and grasslands | 43°931082′N and 85°533013′E | 1581 m.a.s.l. Spruce, shrubs and rich vegetation. |
|  | Shanghudi Township | Wetlands and grasslands | 44°554145′N and 85°896720′E | 375 m.a.s.l. Reeds, narrow-leaved oleaster and rich vegetation. |
| Shihezi | Huayuan Township | Semi-desert and hills | 44°181168′N and 85°788970′E | 788 m.a.s.l. Sparse bush and short grass. |
| Tacheng | Kulusitai Grassland | Grasslands and wetlands | 46°456370′N and 83°344171′E | 451 m.a.s.l. Savanna, reeds, *Achnatherumsplendens* and *halimodendronhalodendron* |
| Yining | Jiliyuzi Township | Grasslands and hills | 44°003681′N and 81°558182′E | 928 m.a.s.l. Diversiform-leaved poplar, willow and rich vegetation. |
