# Supplementary material for: A broad-range survey of ticks from livestock in Northern Xinjiang: changes in tick distribution and the isolation of Borrelia burgdorferi sensu stricto
Source: Parasit Vectors. 2015 Sep 4;8:449. doi: 10.1186/s13071-015-1021-0 (PMC4560164; doi:10.1186/s13071-015-1021-0)
Supplement: Additional file 2: — PCR protocol for the detection of 110 representative tick specimens and Borrelia isolates, northern Xinjiang, China. (DOC 50 kb) [file 13071_2015_1021_MOESM2_ESM.doc]

**Additional file 2**

**PCR protocol for the detection of 110 representative tick specimens and Borrelia isolates, northern Xinjiang, China**

The PCR equipment was a TechneTC-412 thermal cycler, Barloworld Scientific, Cambridge, UK.

**1. PCR amplification of*16S rDNA* mitochondrial gene sequences from 110 representative tick specimens**

Each reaction consisted of 1 μL of tick genomic DNA (50 ng) and 12.5 μL of a PCR mix containing 50 mM KCl, 10 mM Tris-HCl (pH 8.3), 1.5 mM MgCl_2_, 250 μM of each dNTP, 40 pmol of each primer (*16S rDNA*), and 1.0 U of Taq DNA polymerase (TaKaRa Taq Version 2.0, Takara, Dalian, China).The cycling conditions consisted of an initial 5-min denaturation at 94°C, followed by 38 cycles at 94°C for 30 s, 54°C for 30 s, and 72°C for 1 min, with a final extension at 72°C for 8 min.

**2. PCR amplification to detect *Borrelia* isolates based on the *5S-23S rRNA* intergenic spacer**

The PCR amplifications were performed in a 25-μL reaction volume. The reaction mixture contained 0.75 μmol/L of each primer (rrf-O), 250 μM of each dNTP, and 1.0 U of Taq polymerase (TaKaRa Taq Version 2.0, Takara, Dalian, China). The cycling conditions consisted of an initial 5-min denaturation at 94°C, followed by 35 cycles at 94°C for 30 s, 55°C for 30 s, and 72°C for 30 s, with a final extension at 72°C for 8 min.

**3. PCR amplification to detect *Borrelia* isolates based on the outer surface protein A (*OspA*) gene**

The PCR amplifications were performed in a 25-μL reaction volume. The reaction mixture contained 0.75 μmol/L of each primer, 250 μM of each dNTP, and 1.0 U of Taq polymerase (TaKaRa Taq Version 2.0, Takara, Dalian, China). The cycling conditions consisted of an initial 5-min denaturation at 94°C, followed by 35 cycles at 94°C for 30 s, 55°C for 30 s, and 72°C for 1 min, with a final extension at 72°C for 8 min.

Nucleotide sequences of the primers used for the identification of ticks and *Borrelia spp.*

| Gene | Primer | Sequence(5’-3’) | Reference |
| --- | --- | --- | --- |
| *16SrDNA* | 16s-F | CTGCTCAATGATTTTTTAAATTGCTGTGG |  |
|  | 16s-R | CCGGTCTGAACTCAGATCAAGT |  |
| *5S-23S* rRNA | rrf-OF | CGACCTTCTTCGCCTTAAAGC |  |
|  | rrl-OR | TAAGCTGACTAATACTAATTACCC |  |
| *OspA* | OspA-F | CATATGAACAGCGTTTCAGTAGATTTG | This study |
|  | OspA-R | CTCGAG TTTTAAAGCGTTTTTAATT | This study |

References

1. Black WCt, Piesman J: **Phylogeny of hard- and soft-tick taxa (Acari: Ixodida) based on mitochondrial 16S rDNA sequences.** *Proceedings of the National Academy of Sciences of the United States of America* 1994, **91:**10034-10038.

2. Chao LL, Liu LL, Shih CM. Prevalence and molecular identification of Borrelia spirochetes in Ixodes granulatus ticks collected from Rattus losea on Kinmen Island of Taiwan. Parasit Vectors. 2012, 5:167.
